# Identification of candidate genes responsible for chasmogamy in wheat

**DOI:** 10.1186/s12864-023-09252-1

**Published:** 2023-04-04

**Authors:** Magdalena Szeliga, Beata Bakera, Magdalena Święcicka, Mirosław Tyrka, Monika Rakoczy-Trojanowska

**Affiliations:** 1 Rzeszow University of Technology, Powstańców Warszawy 12, 35-959 Rzeszów, Poland; 2 Faculty of Biology, Institute of Experimental Plant Biology and Biotechnology, University of Warsaw, Miecznikowa Street 1, 02-096 Warsaw, Poland; 3 Warsaw University of Life Sciences, Nowoursynowska 166, 02-787 Warsaw, Poland

**Keywords:** Wheat breeding, Cleistogamy, Chasmogamy, Lodicules, RNA-Seq, RDA-Seq

## Abstract

**Background:**

The flowering biology of wheat plants favours self-pollination which causes obstacles in wheat hybrid breeding. Wheat flowers can be divided into two groups, the first one is characterized by flowering and pollination within closed flowers (cleistogamy), while the second one possesses the ability to open flowers during processes mentioned above (chasmogamy). The swelling of lodicules is involved in the flowering of cereals and among others their morphology, calcium and potassium content differentiate between cleistogamic and non-cleistogamous flowers. A better understanding of the chasmogamy mechanism can lead to the development of tools for selection of plants with the desired outcrossing rate. To learn more, the sequencing of transcriptomes (RNA-Seq) and Representational Difference Analysis products (RDA-Seq) were performed to investigate the global transcriptomes of wheat lodicules in two highly chasmogamous (HCH, Piko and Poezja) and two low chasmogamous (LCH, Euforia and KWS Dacanto) varieties at two developmental stages—pre-flowering and early flowering.

**Results:**

The differentially expressed genes were enriched in five, main pathways: “metabolism”, “organismal systems”, “genetic information processing”, “cellular processes” and “environmental information processing”, respectively. Important genes with opposite patterns of regulation between the HCH and LCH lines have been associated with the lodicule development i.e. expression levels of MADS16 and MADS58 genes may be responsible for quantitative differences in chasmogamy level in wheat.

**Conclusions:**

We conclude that the results provide a new insight into lodicules involvement in the wheat flowering process. This study generated important genomic information to support the exploitation of the chasmogamy in wheat hybrid breeding programs.

**Supplementary Information:**

The online version contains supplementary material available at 10.1186/s12864-023-09252-1.

## Background

Common wheat (*Triticum aestivum* L.) is a self-pollinated crop in which pollination and fertilization usually occur before the florets open, which makes pollination with foreign pollen unlikely [1–4]. Self-pollination is ensured via several mechanisms, e.g. cleistogamy [3], the short initial phase of floret opening [5], the restriction of the anthers inside the florets, the low efficiency of anther extrusion [6], and heavy pollen [1, 7]. Moreover, the flowers of wheat do not attract insects or animals for the purpose of pollination as they have no colourful petals, nectar, nor attractive odours.

In cereals, the floret opening at anthesis is caused by the lodicules swelling [1, 6, 8–10]. Lodicules are floral organs with scale-like shapes, specific for grasses [11]. In wheat, the pair of lodicules located between the lemma and the ovary base swell speedily at anthesis, push apart the rigid lemma which allows anthers and stigma to emerge [8, 12]. The process related to turgid lodicules is referred to as ‘first opening’ and usually takes less than 30 min [1, 5, 13, 14]. After this time and probably in response to pollination, the lodicules collapse and the floret closes [8]. After a few days, a process called the ‘second opening’, caused by the enlargement of the unfertilized ovaries which generates the lateral push of the rigid lemma and palea is observed [5].

Lodicules are important to the cereal flowering process. Lodicules of the non-cleistogamous wheat cultivar YM18 show higher calcium and potassium contents, and are morphologically different from the respective cleistogamic mutant line—*ZK001* [15]. The comparative transcriptome analysis of spikelets and lodicules indicated that the main differentially expressed genes between cleistogamous and non-cleistogamous wheat genotypes were related to carbohydrate metabolism, protein transport, phytohormones and calcium ion binding [15, 16]. These genes play an important role in regulating cellular homeostasis, osmotic pressure, and finally lodicule development. Moreover, the genes involved in carbohydrate metabolism and the regulation of potassium/calcium ions are closely associated with water absorption and play an important role in lodicule expansion [12, 17, 18].

Several genes potentially relevant in the context of flower opening function have been isolated. These genes direct lodicule development—TaAP2-A, TaAP2-B, TaAP2-D [19], confer pollen sterility—*TaPaO1* [20], code for tonoplast aquaporins—TIP1, TIP2 [21] or are down-regulated in the unfertilized swelling ovaries—TaVPE4-A, TaVPE4-B, TaVPE4-D (orthologues of barley HvVPE4 involved in pericarp programmed cell death, PCD) [5]. Using a bioinformatic approach, 900 putative orthologs of 190 known *Arabidopsis* flowering-related genes were identified in wheat [22]. The expression of these flowering-related genes was in silico profiled in 13 different developmental stages. The flowering-related genes belonged to one of the seven functional groups, namely: autonomous (including ambient temperature pathway), flower development, gibberellin, photoperiod, pathway integration, regulation, and vernalization. In this pool, genes encoding gibberellin 20 oxidase (Traes_4AL_FABDF4EDA), glucose-6-phosphate isomerase (Traes_5DL_BD1C8E19E) and enzymes from UDP-Glycosyltransferase superfamily protein (Traes_1AL_1966DEE15), 2-oxoglutarate (2OG) and Fe(II)-dependent oxygenase superfamily protein (Traes_7AL_9D1BEACC0) were found. However, their role in controlling wheat flowering is still not fully clarified.

Cleistogamy corresponds to the morphological, physiological and biochemical mechanisms leading to flowering with closed flowers and preventing from anther extrusion and pollen release at least after anthesis [23]. Anthesis of flowering plants includes flower opening and closure, anther dehiscence, and fertilization of the ovule [24]. In grasses at anthesis stage, opening and closing flowers is determined by distance between palea and lemma [25] and is driven by swelling and subsequent withering of the lodicules [5, 12, 24, 26–28]. Cleistogamy may result from poorly developed, smaller, dysfunctional lodicules [12], stiff and large glumes, stiff lemma, and small anthers [5, 29–32]. Alternatively, in barley, cleistogamous genotypes that reach anthesis before the spike has emerged from the boot can be selected. In these forms the lodicules have already begun to shrink by the time of the spikes emerging from the boot, and they are no longer able to push the lemma and palea apart [33]. In wheat, an ovary unfertilized at anthesis stage swells and triggers the ‘second opening’ [5]. The second component of cleistogamy are the interdependent processes of pollen release preceded by stamen filament extension leading to anthers extrusion [12].

The identification of genetic factors for wheat cultivars with different chasmogamy levels contributes to a better understanding of the chasmogamy mechanism and the development of tools for selection of plants with desired outcrossing rate. The high percentage of chasmogamous flowers is a component of the traits set necessary for promoting the outcrossing rate in wheat hybrid seed production. Aside from lodicule properties, other traits significant for hybrid wheat are anther extrusion and retention, the relationship between the ovary/floret size ratio, the flower opening angle, nicking (i.e. optimal flowering coincidence), the amount of pollen [5, 6], and pollen features that determine its mobility. The polyploid nature of hexaploid wheat provides an additional obstacle in conventional breeding cultivars with the desired cleistogamy or chasmogamy level, as functional alleles at each of the three homoeoloci may be necessary to the obtain desired genotype. Marker assisted accumulation of cleistogamous alleles should help in the development of cleistogamous form of bread wheat [34]. Ovary swelling is useful for facilitating cross-pollination in hybrid breeding [5] and due to the double stage flowering in wheat, the properties of lodicules and percentage of flowers closed at “first flowering” seem not to be crucial for the development of female components in hybrid breeding.

In wheat, the pair of lodicules swell and wither leading to glume opening and glume closing at anthesis [35, 36]. These processes are due to the water flow into and out of the lodicule cells associated with the accumulation of K^+^ [12, 16, 37], accumulation and transport of calcium in the epidermis and outer parenchymatous cells of lodicules [15, 16, 18], concentration of soluble sugars [15, 38, 39], starch [15, 40], signalling by jasmonic acid and its derivatives (JAs) [37, 39, 40], and controlled by K + transporters [37, 39], aquaporins [36] or stimulus associated with pollination [8]. In sorghum, exogenous MeJA treatments could induce the up- or down- regulation of genes related to starch and sucrose metabolism, alpha-linolenic acid metabolism and plant hormone signal transduction pathways in the plasma cells of sorghum florets, thereby promoting the opening of sorghum florets [40].

In chasmogamous florets, simultaneous lodicule enlargement and the extension of the stamen filaments result in the synchronized emergence and dehiscence of the anthers, assisting self-pollination. However, the influx of osmoticum into the lodicules and stamen filaments may be controlled independently [12]. Environmental factors influence the anthesis process. In photo-thermo-sensitive genic male sterile (ptGMS) rice, high temperatures during anthesis reduced the percentage of opened spikelets, the spikelet-opening angle, the length of spikelet-opening time, fertilization percentage and seed-setting leading to the grain yield decrease [41]. The high temperature also significantly decreased the contents of soluble sugars, jasmonic acid (JA) and methyl jasmonate (MeJA) in the lodicules before and at glume-opening [41].

Flowering genes can be regulated at transcriptional, posttranscriptional, epigenetic, and posttranslational levels [22]. miRNA may regulate expression of floral organs [22, 25, 32]. In non-cleistogamous barley cultivars, the cleistogamy1 (cly1) mRNA (determine atrophy of lodicule) may be degraded by miR172-directed cleavage [26]. A single-nucleotide substitution in the miR172 target site (allele cly1.b) prevents mRNA cleavage and allows the lodicules to swell [33, 42]. In cleistogamous barley cv. SV235 downregulation of cly1 is caused by an epiallele that represses transcription [42]. Gene *cly1* is an ortholog of the *Arabidopsis thaliana* APETALA2 (AP2) transcription factor [26]. In wheat, no functional mutations at the miR172 targeting site were identified [19].

The wheat floret architecture supporting self-pollination is unfavourable in heterosis breeding. In order to efficiently generate wheat hybrids, the female component must be male sterile, and must show the ability to open flowers called chasmogamy. The male component carries fertility restoration (*Rf*) genes and is expected to efficiently extrude anthers during flowering. The still poorly understood mechanisms underlying flowering in wheat prompted us to undertake research aimed at the identifying genes controlling this process, in particular the ability to floret opening. In order to achieve this goal, the sequencing of transcriptomes (RNA-Seq) and Representational Difference Analysis products (RDA-Seq) was performed to investigate the global transcriptomes of wheat lodicules in two highly-chasmogamous (HCH) (Piko and Poezja) and two low-chasmogamy (LCH) (Euforia and KWS Dacanto) varieties at two developmental stages—pre-flowering and early flowering.

## Materials and methods

### Plant growth conditions and sampling

The plant material consisted of two HCH cultivars: Piko (DE, 2009, CWW-3319.5/3/Kraka//Maris Huntsman/Fruhgold) and Poezja (LT, 2017, STH43/STH248-93–14-326), and two LCH cultivars: Euforia (PL, 2018, Muszelka//N5113/STH3907 and KWS Dacanto (PL, 2011, Opus/Certo) cultivars of hexaploid winter wheat. Kinetics and the micro- morphological and anatomical structures of spikelets in Piko and Dacanto as representatives of HCH and LCH groups were characterized. Both cultivars produce extruded anthers, but Piko showed approximately two-fold higher proportion of extruded anthers compared to the Dacanto (42.2% ± 1.9 vs. 18.6% ± 3.0, respectively) [6].

After 6-week vernalization at 4˚C, the plants were transferred to a growth chamber (ForClean, ZalMed Sp. z.o.o, Warsaw, Poland) and grown as described previously [43]. In order to identify candidate genes controlling lodicules opening, lodicules of HCH and LCH genotypes were harvested from spikes at pre-flowering (GS59—ear completely emerged above flag leaf ligule) corresponding to green anther stage (GAS) and early flowering (GS61—start of flowering (first anthers visible) referred as yellow anther stage (YAS) (5, 32). The samples were collected in three biological replicates. Approximately 50–100 lodicules were samples from five to seven individual spikes for each of the three replicates. The materials were immediately submerged in RNAlater™ Stabilization Solution (Ambion, Thermo Fisher Scientific, Waltham, MA, USA) and kept at -80˚C until required.

### RNA isolation

GeneMATRIX Universal RNA Purification kit (EURx, Gdańsk, Poland) was used to isolate total RNA from the preserved lodicules. RNA concentration was measured by means of a Qubit® 2.0 Fluorometer (Invitrogen, Waltham, MA, USA) and its quality and integrity were verified on BioAnalyzer 2100 (Agilent Technologies, Santa Clara, CA, USA) using Agilent RNA 6000 Nano Kit.

### Transcriptome sequencing (RNA-seq) and data processing

In total, twenty four strand-specific cDNA libraries (for four cultivars, two anthers developmental stages, and three biological replications) were constructed with NEBNext® Ultra™ II Directional RNA Library Prep Kit for Illumina® (New England Biolabs, Ipswich, MA, USA). The Illumina HiSeq 4000 platform in the PE150 mode was used for RNA-Seq and the data were analyzed by Genomed S.A (Warsaw, Poland).

The first stage of the analysis was removing the adapters using the Cutadapt program. Cutadapt program was in paired-end mode, removing adapters only from side 3' and also polyA sequences (and analogously formed tails with the sequence "T" as well). Additionally, the parameter was also used for removing excessively short readings, where 36 base pairs were adopted as the threshold. Then the readings were mapped by TopHat [44] to the *Triticum aestivum* reference genome with the GenBank number GCA_900519105.1. TopHat was started with the option to prepare the fr-firststrand library and in the no-novel-juncs mode. Later the number of pairs of readings mapped to individual genes were counted using the HTseq program [45] with differentiation due to the transcript strand (–stranded = reverse).

The final results were processed in the R environment using the DESeq2 package for differential expression analysis. Using the biomaRt package, GO identifiers [46] of the analysed genes were collected. Singular Enrichment Analysis (SEA) was performed using default parameters such as Fisher as a statistical test method; Yekutieli (FDR under dependency) as a multi-test adjustment method, and the significance level was set at *p* < 0.05 by on-line tool AgriGO [47]. For the KEGG pathway analysis of the differentially expressed genes (DEGs), KEGG Automatic Annotation Server (KAAS) [48] was used. Venn diagrams of up- and down-regulated genes were generated using jvenn tool [49]. The DEGs were identified by comparing the HCH (Piko and Poezja) and LCH (KWS Dacanto and Euforia) varieties individually and in pairs at the YAS and GAS. DEGs were selected with a FDR < 0.01 and log2 fold change >|-1.99 or 1,99|. Another condition was participation in biological and/or molecular processes related to flowering based on GO analysis. The expression heatmap of DEGs was created using Heatmapper [50]. Most of DEGs were of high quality with more than 100 normalized counts and over 95% of DEGs coded proteins. Additionally, p-value and -log10 (p-value) ranked from 0 to 0.2 and 0 to 1, respectively.

### RDA-Seq and data processing

Representational difference analysis was performed on high quality cDNA [51, 52]. Lodicules at the GAS were used as testers while the lodicules at the YAS were selected as driver probes. This procedure results in the accumulation of unique sequences upregulated in GAS. The analysis was taken in three replicates for each variety (Piko, Poezja, Euforia, and KWS Dacanto). The tester:driver ratio in three subsequent rounds of subtractive hybridization increased from 1:50, and 1:400 to 1:200,000. Difference products obtained from the third subtraction in the size range from 200 to 800 bp were sequenced. Nextera DNA Flex Library Prep Kit (Illumina, San Diego, CA, USA) was used for library construction according to the manufacturer instructions. The cDNA library products were sequenced by paired-end sequencing technology (2 × 300 cycles) on the MiSeq (Illumina, San Diego, CA, USA) platform.

Raw reads were filtered to obtain clean reads by removing read-through adapter sequences, low quality sequences and ambiguous nucleotides. Next, high-quality clean reads were mapped to the *Triticum aestivum* reference genome with the GenBank number GCA_900519105.1 by CLC Genomics Workbench 12 (Qiagen, Germantown, MD, USA). The FPKM (fragments per kilobase of transcript per million mapped) parameter was used to calculate gene expression. In RDA analysis the DEGs were selected with the threshold false discovery rate (FDR) of < 0,05 and the absolute value of log2FoldChange > 1. GO annotation of the DEGs was made by Blast2GO program (BioBam Bioinformatics, Valencia, Spain) and Venn diagram was prepared with jvenn tool [49]. Fasta sequences of DEGs were retrieved from wheat IWGSC cDNA database and annotated with Mercator4 v4.0 [53] sequence annotation pipeline (http://mapman.gabipd.org/home). Expression levels of selected DEGs were validated using WheatOmics platform [54]. Both RDA-Seq and RNA-Seq sequencing data were deposited in the ArrayExpress database via Annotare (https://www.ebi.ac.uk/arrayexpress accessed on 14th November 2022) under number E-MTAB-12139 and E-MTAB-12136.

### Validation of transcripts by RT-qPCR analysis

Relative expression levels of genes selected based on the results of RNA-Seq and RDA-Seq analyses were validated by RT-qPCR (reverse transcription quantitative real-time PCR) assays as described previously [43]. Fragments of selected cDNA sequences were retrieved from RDA-Seq contigs and used for gene specific primer design (Table S8). *HvAct* (GenBank, accession no. AY145451) was used as a reference gene in RT-qPCR reactions. The significance of the differences between ΔCt values was calculated by Mann–Whitney U test (with continuity correction) in STATISTICA 12 package (TIBCO Software, USA).

## Results

### General analysis of RNA-Seq data

Sequencing of 24 RNA-Seq libraries resulted in 891 465 223 raw reads, and a number of reads varied between 28,9 to 44,3 million per library. A total of 890 180 463 high quality reads were filtered out and 683 683 129 (76.8%) were mapped to the *Triticum aestivum* reference genome (GenBank number GCA_900519105.1).

### DEGs identified in RNA-Seq analysis

In order to identify DEGs in HCH (Piko and Poezja) and LCH (KWS Dacanto and Euforia) varieties, genes expression levels were compared separately for the GAS and YAS resulting in eight comparisons C1-C8 (Table 1). At the GAS, a total of 2961, 2265, 4353 and 2216 DEGs were up- or downregulated in CHC vs LCH varieties defined as sets C1, C3, C5 and C7, respectively (Table 1). At the YAS, the number of DEGs varied from 990 to 1566 and from 928 to 1076 for upregulated and downregulated DEG, respectively. In total, 4131 and 4576 genes were identified as upregulated in the GAS and YAS and 7664 and 4091 genes were identified as downregulated in the GAS and YAS, respectively (Table 1).

**Table 1:** DEGs identified in the RNA-Seq analysis. Number of up- and downregulated genes in HCH varieties (Piko and Poezja) vs LCH (KWS Dacanto and Euforia) controls in the GAS and YAS stages

Comparison No	Stage and Compared Cultivars	Number of selected DEGs	
		Upregulated	Downregulated
C1	YAS: Piko/KWS Dacanto	850	2111
C2	GAS: Piko/KWS Dacanto	1024	928
C3	YAS: Piko/Euforia	1200	1065
C4	GAS: Piko/Euforia	1566	1076
C5	YAS: Poezja/KWS Dacanto	955	3398
C6	GAS: Poezja/KWS Dacanto	990	1072
C7	YAS: Poezja/Euforia	1126	1090
C8	GAS: Poezja/Euforia	996	1015

**Fig. 1:**
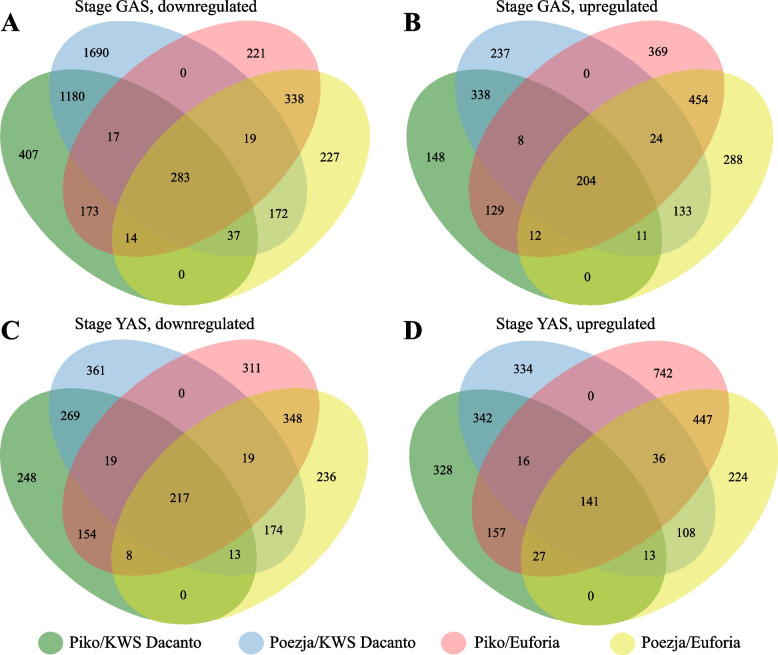
Venn diagrams indicating the number of overlapping down- and upregulated genes in the GAS and YAS in the C1-C8 comparisons defined in Table 1. The number of genes downregulated (**A**, **C**) and upregulated (**B**, **D**) between HCH and LCH varieties in the YAS (**A**, **B**) and GAS (**C**, **D**) stages

**Fig. 2:**
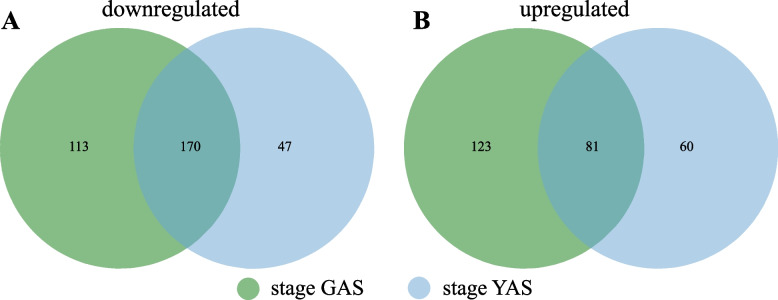
Venn diagram showing the total number of downregulated (**A**) and upregulated (**B**) transcripts in HCH vs LCH varieties in the GAS and YAS

DEGs selected for pairwise comparisons were further analyzed in order to identify genes commonly up- or downregulated at the GAS and YAS. The highest number of unique DEGs was observed for genes downregulated in Poezja vs KWS Dacanto at the GAS stage, and for genes upregulated in Piko vs Euforia at the YAS stage. In these combinations unique genes accounted for 47%-49% of all DEGs. In remaining comparisons from 17.4% to 33.7% genes were unique. Assuming that the mechanism responsible for chasmogamous flowering in Piko and Poezja is similar, common genes regulating this process should be identified. A total of 500 and 326 genes were commonly down- and upregulated in two analyzed stages for four pairs of genotypes compared (Fig. 1). Next, the selected DEGs showing the same trend in chasmogamous varieties were analysed for flower developmental stage specific expression. In total, 330 downregulated and 264 upregulated DEGs were found (Table S1). Up- and downregulated stage independent transcripts accounted for 30% and 51%, respectively. These genes are possibly responsible for processes accompanying flower opening. Remaining 160 downregulated and 183 upregulated stage specific genes may play a key role in flower opening (Fig. 2).

### Functional classification of DEGs in RNA-Seq analysis

**Fig. 3:**
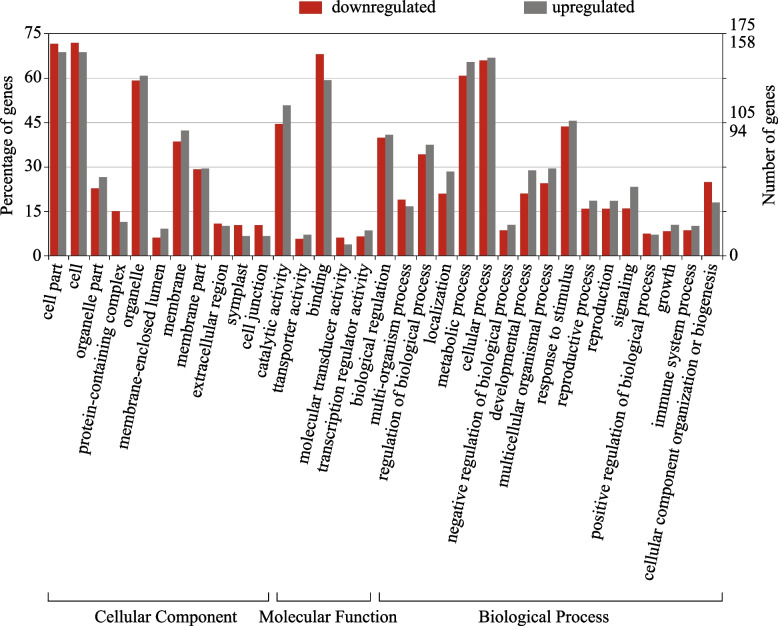
GO classification of non-redundant DEGs down- and upregulated in chasmogamus varieties identified in RNA-Seq analysis

GO analysis was applied to investigate the function of the selected DEGs down- and upregulated in HCH vs LCH varieties. GO annotations were available for the 234 (70.9%) and 211 (79.9%) of down- and upregulated DEGs, respectively. The GO terms were distributed over 56 significant functional groups in three broad categories of ontologies comprising ‘biological process’ (BP), ‘molecular function’ (MF), and ‘cellular component’ (CC). The BP category of GO was mainly represented by ‘cellular process’ (GO:0,009,987), ‘metabolic process’ (GO:0,008,152) and ‘response to stimulus’ (GO:0,050,896). The ‘cell’ (GO:0,005,623), ‘cell part’ (GO:0,044,464) and ‘organelle’ (GO:0,043,226) had the greatest number of genes in the CC category. The molecular function category of GO was mainly sub-categorized by ‘binding’ (GO:0,005,488) and ‘catalytic activity’ (GO:0,003,824), (Fig. 3).

**Fig. 4:**
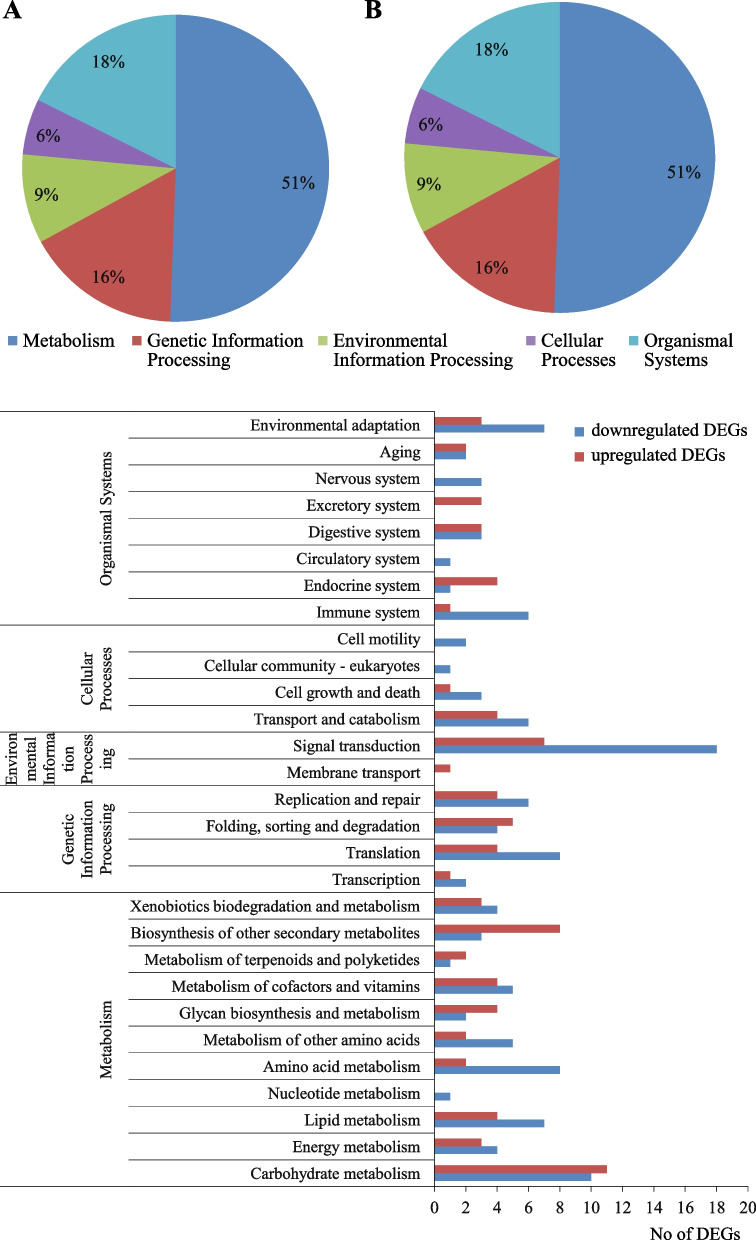
KEGG classifications of selected DEGs up-(**A**) and downregulated (**B**) in HCH varieties

KEGG pathways enrichment analysis was employed to study the major biological pathways that involved the selected DEGs. Only 81 and 103 up- and downregulated DEGs were assigned to five main KEGG categories. The most of DEGs was classified into “metabolism”, “organismal systems”, and “genetic information processing”. Less represented were “cellular processes”, and “environmental information processing” (Fig. 4).

More detailed KEGG classification showed that the downregulated DEGs were connected mainly with signal transduction, environmental adaptation, translation, amino acid, carbohydrate and lipid metabolism. Upregulated DEGs dominated in biosynthesis of other secondary metabolites and carbohydrate metabolism categories (Fig. 4).

### Enrichment analysis

GO enrichment analysis (SEA) was utilized to identify GO terms related to flower development significantly up- or down-regulated in sets of DEGs. Within the 234 downregulated DEGs no significantly enriched GO terms were found. Analysis of 211 upregulated genes revealed nine significant GO terms in categories BP and MF. In BP category, 20 unique DEGs were characterized with multiple GO terms. From 9 to 19 of the DEGs were assigned to the selected GO term that included ‘stamen filament development’, ‘regulation of organ growth’, ‘organ growth’, ‘auxin polar transport’, ‘hormone transport’, ‘auxin transport’, and ‘regulation of developmental growth’. In MF ontology class, two GO terms (‘aspartic-type endopeptidase activity’ and ‘aspartic-type peptidase activity’) were simultaneously identified in seven genes (Table 2, Figure S1).

**Table 2:** GO terms selected in SEA analysis of DEGs upregulated in chasmogamous varieties

GO Term	Ontology	Description	No of DEGs	*p*-value	FDR
GO:0,080,086	BP	stamen filament development	9	8.0e-10	2.8e-06
GO:0,046,620	BP	regulation of organ growth	12	7.5e-08	0.00013
GO:0,035,265	BP	organ growth	12	1.6e-07	0.00018
GO:0,009,926	BP	auxin polar transport	11	3.5e-06	0.003
GO:0,009,914	BP	hormone transport	11	1.0e-05	0.0058
GO:0,060,918	BP	auxin transport	11	9.4e-06	0.0058
GO:0,048,638	BP	regulation of developmental growth	19	6.6e-05	0.032
GO:0,004,190	MF	aspartic-type endopeptidase activity	7	0.0002	0.046
GO:0,070,001	MF	aspartic-type peptidase activity	7	0.0002	0.046

### Characterization of DEGs identified in GO enrichment analysis

**Fig. 5:**
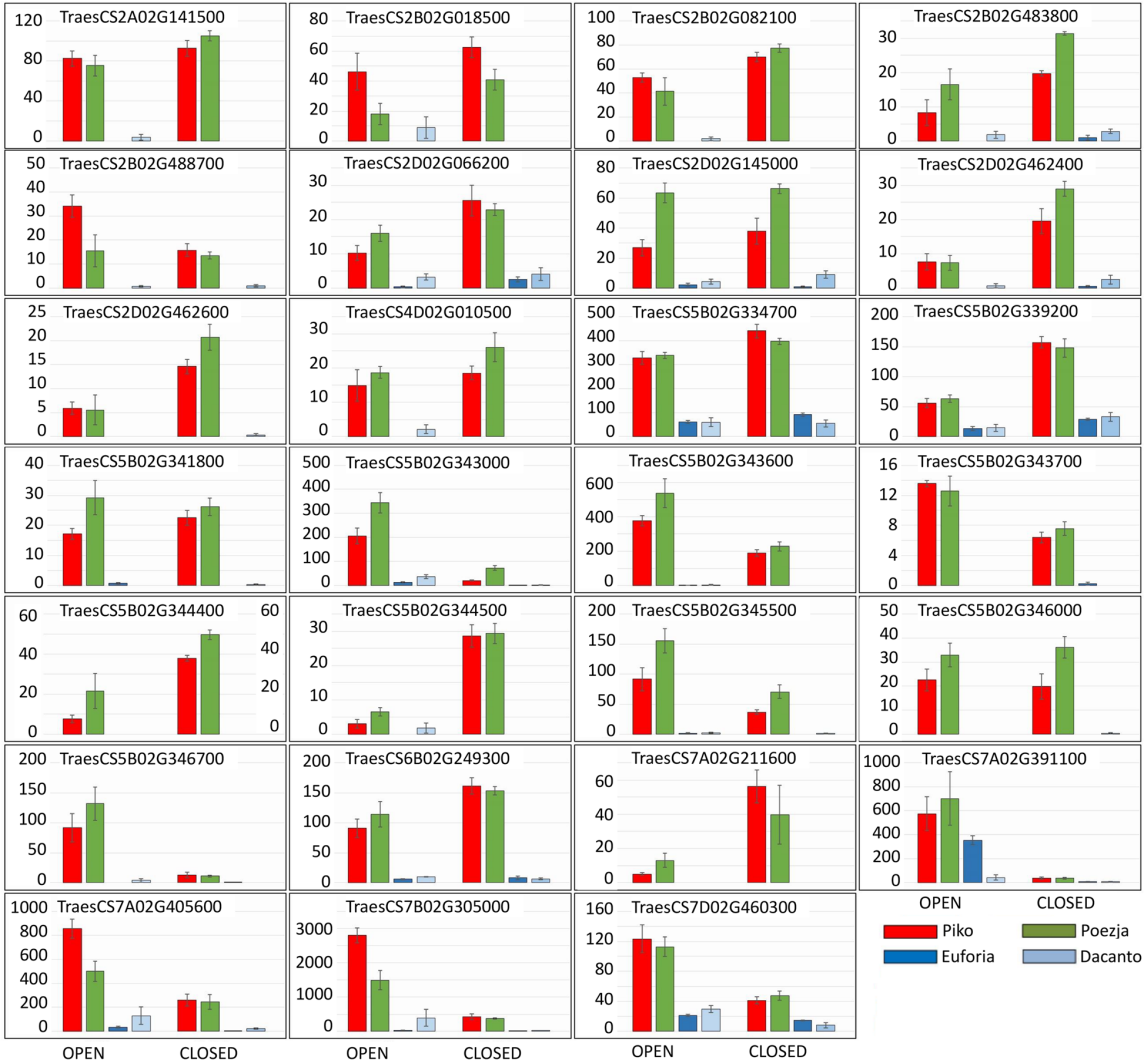
Expression pattern of 27 DEGs selected in GO enrichment analysis

In result of GO enrichment analysis, all DEGs downregulated in chasmogamous varieties were dropped and only upregulated DEGs were selected. We found 27 DEGs simultaneously upregulated in HCH varieties ‘Piko and Poezja (Table 3). Only two DEGs were selectively upregulated at the YAS stage (TraesCS5B02G343700, and TraesCS7D02G460300). 8 DEGs (TraesCS2B02G082100, TraesCS5B02G334700, TraesCS5B02G341800, TraesCS5B02G343000, TraesCS5B02G343600, TraesCS5B02G345500, TraesCS5B02G346000, and TraesCS6B02G249300) were upregulated in the both stages (Table 3, Fig. 5). All remaining DEGs were upregulated at the GAS stage (Table 3, Fig. 5, Tables S1 and S2).

**Table 3:** Best BLASTx hits for 27 DEGs selected by a RNA-Seq analysis

Gene symbol	Stage	Protein function	NCBI accession
**Auxin responsive**			
TraesCS5B02G341800	GAS/YAS	Auxin responsive SAUR36-like	XM_044534055.1
TraesCS5B02G343000	GAS/YAS	Auxin responsive SAUR36-like	XM_020315849.3
TraesCS5B02G343600	GAS/YAS	Auxin responsive SAUR36-like	XM_037587073.1
TraesCS5B02G343700	YAS	Auxin responsive SAUR36-like	XM_045229117.1
TraesCS5B02G344400	GAS	Auxin responsive SAUR36-like	XM_044534070.1
TraesCS5B02G344500	GAS	Auxin responsive SAUR36-like	XM_045128340.1
TraesCS5B02G345500	GAS/YAS	Auxin responsive SAUR36-like	XM_044537684.1
TraesCS5B02G346000	GAS/YAS	Auxin responsive SAUR36-like	XM_044537687.1
TraesCS5B02G346700	GAS	Auxin responsive SAUR36-like	XM_044534083.1
TraesCS7A02G391100	GAS	ABC transporter G family member 44-like	XM_044567717.1
**Stress related**			
TraesCS2B02G018500	GAS	putative disease resistance protein RGA3 isoform X1, X2	XM_044465387.1
TraesCS2B02G082100	GAS/YAS	probable LRR receptor-like serine/threonine-protein kinase At3g47570	XM_044465869.1
TraesCS2B02G488700	GAS	yellow rust resistance protein 5a	MN273772.1
TraesCS2D02G066200	GAS	probable LRR receptor-like serine/threonine-protein kinase At3g47570	XM_044474111.1
TraesCS5B02G339200	GAS	rop guanine nucleotide exchange factor 1-like (floral organ development)	XM_037587057.1
TraesCS7A02G211600	GAS	subtilisin-like protease SBT1.8	XM_044569899.1
TraesCS7D02G460300	YAS	inorganic pyrophosphatase 2-like (salt stress)	XM_044584582.1
**Mitosis**			
TraesCS2B02G483800	GAS	desmethyl-deoxy-podophyllotoxin synthase-like	XM_044464621.1
TraesCS2D02G462400	GAS	desmethyl-deoxy-podophyllotoxin synthase-like	XM_044472898.1
TraesCS2D02G462600	GAS	desmethyl-deoxy-podophyllotoxin synthase-like	XM_044472899.1
TraesCS6B02G249300	GAS/YAS	enhancer of rudimentary homolog isoform X1	XM_044557205.1
**Flower development**			
TraesCS4D02G010500	GAS	signal peptide peptidase-like 4	XM_037598858.1
TraesCS5B02G334700	GAS/YAS	aspartyl protease family protein At5g10770-like (gluten associated)	XM_044534001.1
TraesCS7A02G405600	GAS	aspartyl protease family protein At5g10770-like (gliadin hydrolysis)	XM_044567918.1
TraesCS7B02G305000	GAS	aspartyl protease family protein At5g10770-like	XM_044575753.1
**Unknown**			
TraesCS2A02G141500	GAS	hypothetical protein CFC21_015763	XM_044474837.1
TraesCS2D02G145000	GAS	uncharacterized protein	XM_044474839.1

Annotations with blastn search revealed that the selected DEGs can be classified into genes participating in auxin signalling, general stress reaction, regulation of mitosis, and developmental processes. Nine auxin responsive genes (TraesCS5B02G341800, TraesCS5B02G343000, TraesCS5B02G343600, TraesCS5B02G346700, TraesCS5B02G344400, TraesCS5B02G344500, TraesCS5B02G345500, TraesCS5B02G346000, and TraesCS5B02G343700) were involved in the negative regulation of organ growth, auxin synthesis and transport. These genes were physically clustered at 1.98 Mbp region located at 526–528 Mbp on chromosome 5B (Table S3). In Arabidopsis, these genes act as a positive regulators of leaf senescence and may mediate auxin-induced leaf senescence [55], play a role in the regulation of seed germination by gibberellins and abscisic acid (ABA), and in the regulation of light-dependent hypocotyl elongation [56]. Expression pattern in wheat lodicules of HCH and LCH varieties suggests its different role during flowering. Most of auxin responsive genes were upregulated in HCH varieties in both the GAS and YAS. TraesCS5B02G343700 was upregulated in the YAS, and TraesCS5B02G344400, TraesCS5B02G344500, and TraesCS5B02G346700 were upregulated in the GAS. Another gene in this group (TraesCS7A02G391100) was reported to mediate the transport, regulate auxin homeostasis and responses influencing cotyledons, roots and root hairs development [57–60].

The second group of DEGs consists of seven genes associated with abiotic and biotic stress response and signalling, including disease resistance. DEGs TraesCS2B02G082100 and TraesCS2D02G066200 were involved in cell to cell signalling through phosphorylating serine/threonine residues. TraesCS7D02G460300 activity is essential for correct regulation of mitochondrial membrane potential, mitochondrial organization and function [61] (Table 3).

The third group includes genes regulating mitosis important in growth and developmental processes. Products of three genes (TraesCS2B02G483800, TraesCS2D02G462400, and TraesCS2D02G462600) supress topoisomerase II, an enzyme being a part of regulatory checkpoints at the entry and progression of mitosis [62] are antagonistic to TraesCS6B02G249300 coding for enhancer of rudimentary homolog isoform X1, playing an essential role in the progression of mitosis (Table 3).

The next one is a group of four DEGs that codes for proteins controlling flower development processes. TraesCS4D02G010500 codes for intramembrane-cleaving aspartic protease (I-CLiP) that plays a critical role in the development and function of the reproductive tissues, especially in pollen development. In *Arabidopsis* aspartic proteases (TraesCS5B02G334700, TraesCS7A02G405600 and TraesCS7B02G305000) are expressed during flowering stage i.e. petal differentiation and expansion stage.

### General and functional analysis of RDA-Seq data

**Fig. 6:**
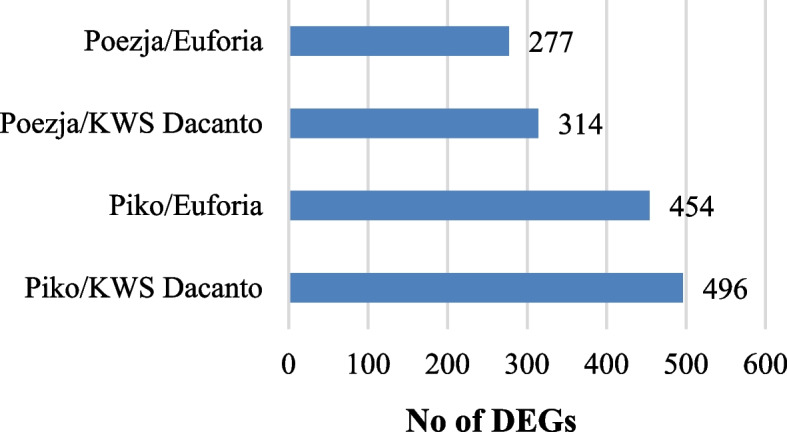
Numbers of differentially expressed genes obtained in RDA-Seq analysis

**Fig. 7:**
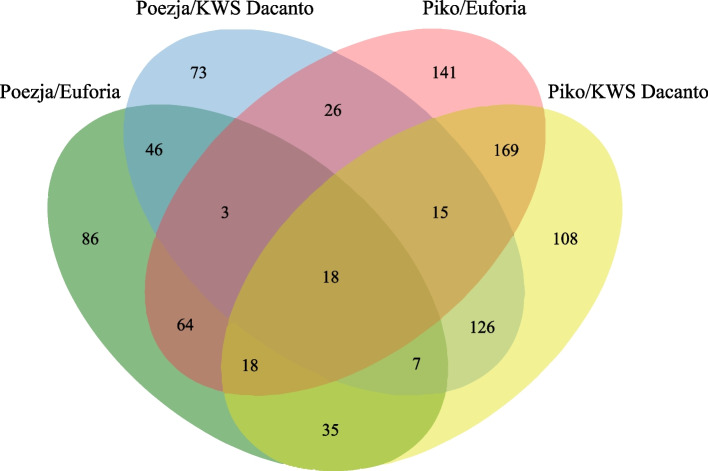
Venn diagram of the number of DEGs for RDA-Seq comparisons

Sequencing of 12 RDA-cDNA libraries obtained after three rounds of subtractive hybridization (RNA from lodicules in the GAS stages were used as testers while lodicules in the YAS—as driver probes) resulted in 21 972 050 raw reads (approximately 10,5 Gb of total nucleotides), ranging from 1,3 to 2,7 million per each library. After removal of adaptor sequences, ambiguous nucleotides and low quality sequences, 31 819 336 high quality reads were obtained and mapped to the *Triticum aestivum* reference genome. The transcriptomes of HCH and LCH varieties were compared. The highest number of 496 and 454 DEGs was observed for Piko HCH variety vs KWS Dacanto and Piko vs Euforia, respectively. The lower number of 314 and 277 DEGs was found in comparisons of Poezja with KWS Dacanto and Euforia, respectively (Fig. 6). The Venn diagram showed the 18 common DEGs for all comparisons RDA-Seq data, representing potential genes controlling flowering (Fig. 7).

**Fig. 8:**
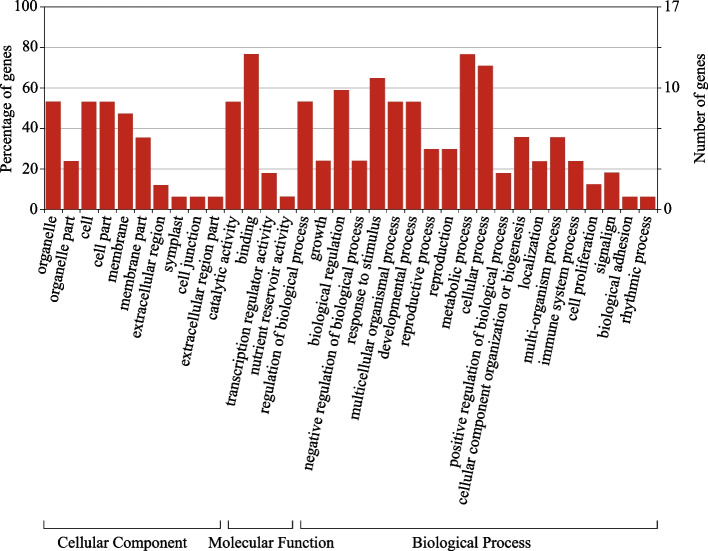
GO classification of 17 DEGs selected in RDA-Seq analysis

A total of 17 predicted transcripts were assigned GO annotations in three main GO categories and 39 subcategories (Fig. 8). The Biological Process category of GO is mainly enriched with ‘cellular process’ (12 genes), ‘metabolic process’ (13 genes) and ‘response to stimulus’ (11 genes) representing 70%, 76% and 64% of total transcripts involved in this category. The ‘cell’ and ‘cell part’ and ‘organelle’ (8 genes) had the greatest number of genes in the cellular component category. The molecular function category of GO was mainly sub-categorized by ‘binding’ (13 genes) and ‘catalytic activity’ (9 genes, Fig. 8).

**Fig. 9:**
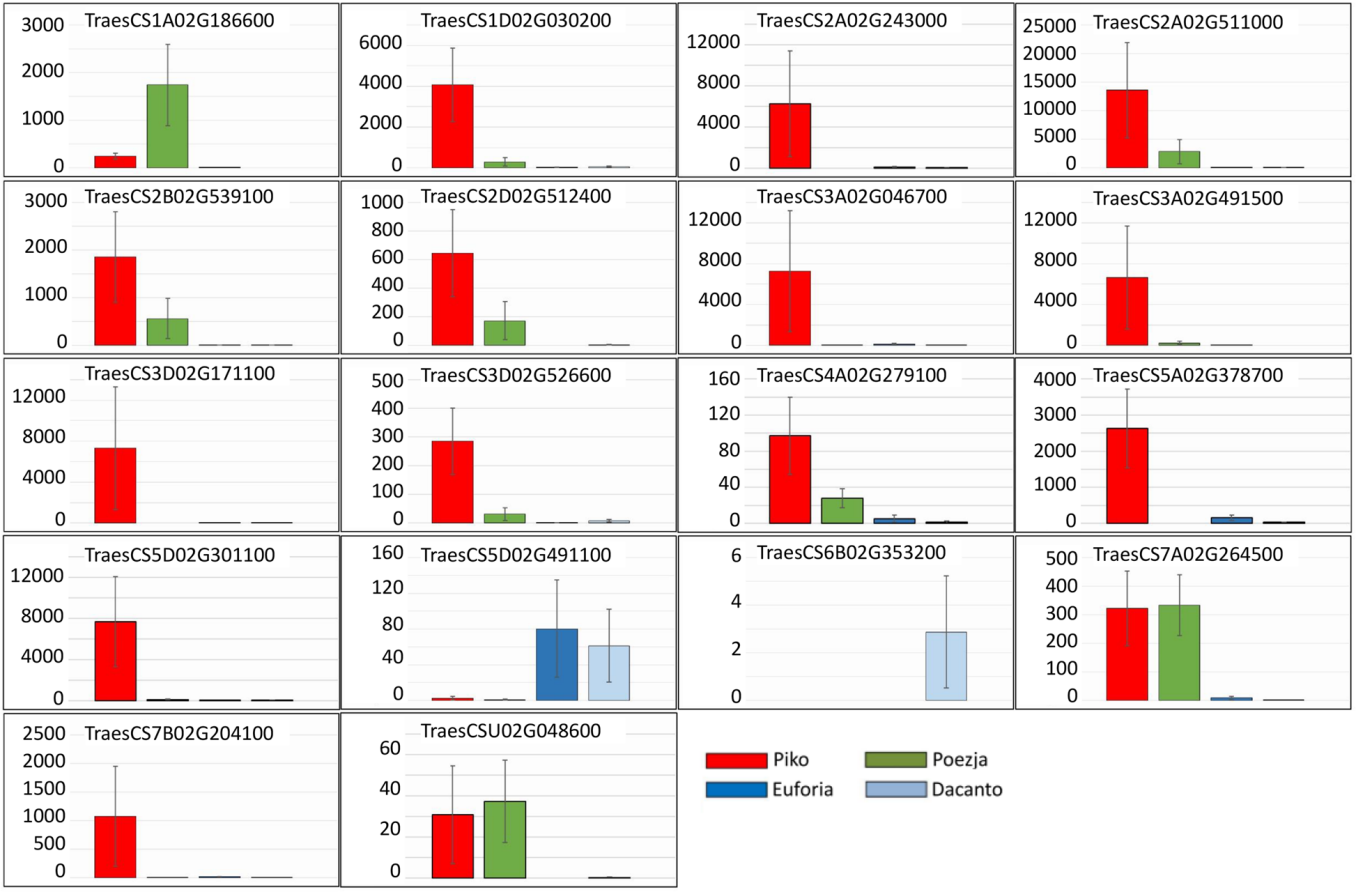
Expression levels (normalized counts) of 18 transcripts selected upon sequencing of RDA products present in the GAS of HCH and LCH varieties

In RDA-Seq analysis, the genes unique to the GAS stage were selected. The majority of these genes were stably overexpressed in both HCH cultivars Piko and Poezja. However, the level of expression of these was usually higher in Piko (Fig. 9, Tables S2 and S3). High and stable expression in HCH cultivars was found for TraesCS7A02G264500 and TraesCSU02G048600. Five DEGs representing genotype specific changes in lodicules (TraesCS2A02G243000.1, TraesCS3A02G046700.1, TraesCS3D02G171100.1, TraesCS5A02G378700.1, TraesCS7B02G204100.1) were upregulated in comparisons with Piko and downregulated in Poezja. Finally, TraesCS5D02G491100.1 was upregulated to high levels in LCH cultivars, and TraesCS6B02G353200 was expressed in KWS Dacanto. DEGs with high and stable expression in cultivars with different intensity of chasmogamy are good candidates responsible for lodicule mediated flower opening.

Based on UniProt characteristics, eight DEGs identified with the RDA-Seq (TraesCS1A02G186600, TraesCS3D02G526600, TraesCS5D02G491100, TraesCS4A02G279100, TraesCS5A02G378700, TraesCS6B02G353200, TraesCS5D02G301100, and TraesCS7A02G264500) were related to stress response and signalling. Among them, TraesCS5D02G491100 codes for C2H2 zinc finger protein, a master regulator of abiotic stress responses that play many roles in plant growth and development [63] was downregulated in HCH cultivars. TraesCS5A02G378700 belongs to plant lipoxygenases and may be involved in jasmonic acid signaling, growth and development [64].

Four another DEGs were annotated as significant for development. Tetraketide alpha-pyrone reductase 2 (TraesCS3A02G046700) of *Arabidopsis* was active in pollen exine formation and sporopollenin biosynthetic processes [65]. TraesCS3A02G491500 regulates transcription and, as a member of SHORT INTERNODES family, regulates tillering and panicle branching in rice [66]. TraesCS6B02G353200 is involved in jasmonic acid synthesis [67]. Monocopper oxidase-like proteins (TraesCS3D02G171100) may be involved in directional growth processes, possibly by participating in cell wall expansion [68]. The remaining genes were involved in secondary metabolite biosynthesis (TraesCS2A02G243000, TraesCS7B02G204100 and TraesCSU02G048600), or coded for proteins not characterized so far (Table 4).

**Table 4:** Characteristics of 18 DEGs selected by a RDA-Seq analysis using the Ensembl Plants database. Upregulation, downregulation and mixed expression in HCH cultivar in comparisons Poezja/Euforia, Poezja/Dacanto, Piko/Euforia, and Piko/Dacanto was marked 4UP, 4DOWN and 2D2U, respectively

Gene symbol	Protein function	Expression	NCBI accession
**Biotic or abiotic stress related**			
TraesCS1A02G186600	probable glutathione S-transferase GSTU6	4UP	XM_044472364.1
TraesCS3D02G526600	disease resistance protein PIK6-NP-like isoform X2	4UP	XM_044503895.1
TraesCS4A02G279100	oxalate oxidase GF-2.8-like	4UP	XM_044509164.1
TraesCS5A02G378700	putative linoleate 9S-lipoxygenase 3	2D2U	XM_044526899.1
TraesCS5D02G301100	uncharacterized LOC123125137 (IPR008480 domain), mRNA	4UP	XM_044545667.1
TraesCS5D02G491100	C2H2 zinc finger protein/ ZAT8-like zinc finger protein	4DOWN	XM_044544001.1
TraesCS7A02G264500	uncharacterized protein LOC123152047 (IPR011692 domain)	4UP	AK454492
**Development**			
TraesCS3A02G046700	tetraketide alpha-pyrone reductase 2-like	2D2U	XM_044480598.1
TraesCS3A02G491500	protein LATERAL ROOT PRIMORDIUM 1-like	4UP	XM_044487267.1
TraesCS3D02G171100	monocopper oxidase-like protein	2D2U	XM_020297783.3
TraesCS6B02G353200	putative 12-oxophytodienoate reductase II	4UP	XM_044558341.1
**Secondary metabolites**			
TraesCS2A02G243000	cytochrome P450 89A2-like	2D2U	XM_044597196.1
TraesCS7B02G204100	cytochrome P450 89A2-like	2D2U	XM_044576729.1
TraesCSU02G048600	indole-2-monooxygenase-like (LOC123064714), mRNA	4UP	XM_044488130.1
**Unknown**			
TraesCS1D02G030200	uncharacterized protein LOC123131461	4UP	XR_006464182.1
TraesCS2A02G511000	uncharacterized protein LOC123042398	4UP	XM_044598116.1
TraesCS2B02G539100	uncharacterized protein LOC123042398	4UP	XM_044464862.1
TraesCS2D02G512400	uncharacterized protein LOC123050360	4UP	XM_044473166.1

### In silico analysis of promoters of selected DEGs

In the 2000 nt upstream sequences of 45 DEGs (Table S4, suppl. materials), 94 cis-regulatory elements (*cre*) were found (Table S5, suppl. materials). Besides the commonly present (CAAT-box, TATA-box), among identified *cre* several categories were distinguished, namely related to light response, stresses, and hormone response, growth and development, tissue and organ specific, and multifunctional *cre* (plant development, secondary metabolism, signal transduction, disease resistance, stress response). The *cre* with the highest abundance exceeding 100 were: ABRE (abscisic acid and hormone responsiveness), CGTCA-motif (MeJA and hormone responsiveness), G-box (light responsiveness), MYB (development, secondary metabolism, signal transduction, disease resistance, and stress response), MYC (plant growth and development, hormone and abiotic stress response), TGACG-motif (MeJA and hormone responsiveness), as-1 (oxidative stress, salicylic acid and auxin response) and STRE (stress response).

Apart from the highly abundant *cre*, 14 *cre* were unique and appeared only once. Among these unique elements, five *cre* were related to light response. Only 3 *cre* (AAGAA-motif, GC-repeat, NON) have no function assigned. The average frequency of *cre* in promoters of DEGs from RNA-Seq and RDA-Seq pools was similar and it was 95 and 98, respectively. The highest number of *cre* (124), was found in TraesCS5B02G346000 (RNA-Seq pool) and the lowest one, (57) – in TraesCS6B02G353200 (RDA-Seq pool).

### Expression of the selected genes determined by RT-qPCR

The expression of 18 selected wheat transcripts was analysed with RT-qPCR. The panel of tested genes included representation of DEGs found in RNA-seq (TraesCS2A02G141500, TraesCS2D02G462400, TraesCS5B02G334700 TraesCS5B02G339200, TraesCS5B02G343000, TraesCS5B02G344400, TraesCS7A02G391100, and TraesCS7A02G405600), and RDA-seq analyses (TraesCS2A02G243000, TraesCS3A02G046700, TraesCS4A02G279100, TraesCS5D02G491100, TraesCS6B02G353200). Additionally, the expression levels of *TaMADS1* (TraesCS5A02G286800), wheat orthologues of *OsMADS58* (TraesCS1A02G125800, TraesCS1B02G144800), and *OsMADS16* (TraesCS7A02G383800, TraesCS7B02G286600) were tested (Table S8).

**Fig. 10:**
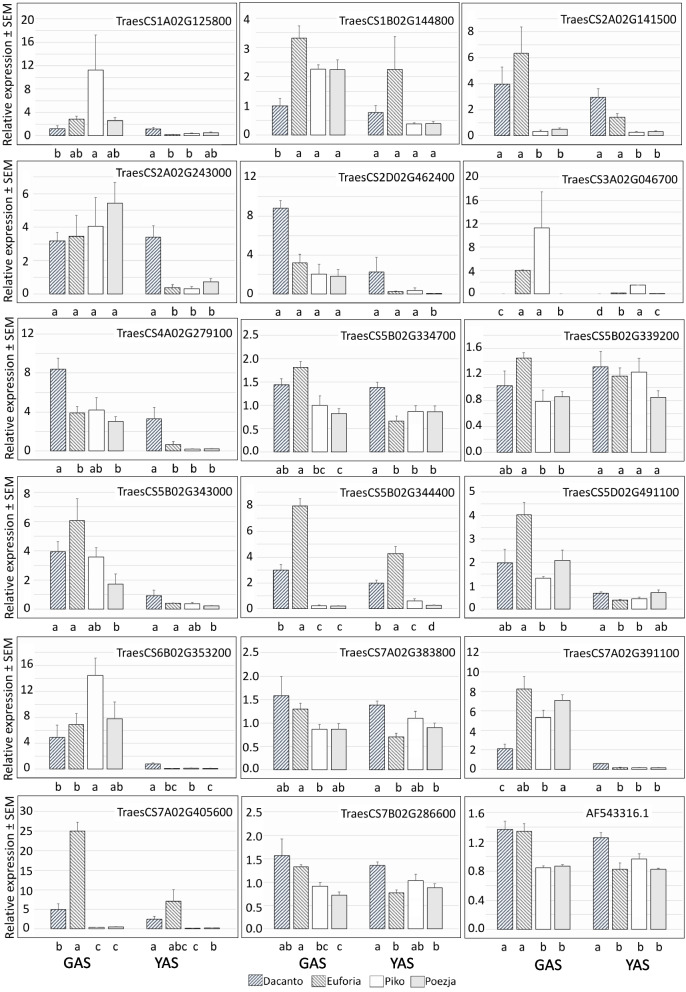
Changes in the expression of 18 genes determined by RT-qPCR

RT-qPCR analyses of TraesCS5B02G343000, TraesCS5D02G491100, TraesCS6B02G353200 and TraesCS7A02G391100 showed higher expression level in the GAS than in the YAS stage. This trend was also maintained for the TraesCS2A02G243000, TraesCS2D02G462400, TraesCS3A02G046700 and TraesCS4A02G279100 genes, although the reaction was more cultivar-specific. TraesCS5B02G334700, TraesCS5B02G339200, TraesCS7A02G383800 and TraesCS7B02G286600 genes showed increased expression in LCH cultivars in the GAS (Fig. 10).

The expression of the most of DEGs selected in RNA-Seq analysis was inconsistent with the qRT-PCR data (Figs. 5 and 10). Generally, qPCR analysis indicated an increased levels of the tested transcripts in the LCH cultivars (i.e. TraesCS2A02G141500, TraesCS2D02G462400, TraesCS5B02G344400, and TraesCS7A02G405600), while quite opposite trends dominated in RNA-Seq data. However, differences in expression levels between the GAS and YAS stages were more consistent and for TraesCS5B02G343000, TraesCS7A02G391100, and TraesCS7A02G405600 similar result were obtained for qPCR and RNA-Seq data. The gene expression profiles of three DEGs selected from RDA-Seq analysis (TraesCS2A02G243000, TraesCS3A02G046700 and TraesCS5D02G491100) relatively agreed with qPCR data (Figs. 9 and 10). However, the expression levels of two other DEGs (TraesCS4A02G279100 and TraesCS6B02G353200) differed substantially between the RDA-Seq and qRT-PCR analyses.

The selected orthologues of *OsMADS58* showed a higher expression in GAS, with genotype dependent pattern. Whereas, two *OsMADS16* orthologues (TraesCS7A02G383800 and TraesCS7B02G286600) showed similar expression level in the GAS and YAS. However, expression of both genes was increased in LCH cultivars in the GAS. This pattern of expression was found also for* TaMADS1* gene (Fig. 10).

## Discussion

### Qualitative cleistogamy and quantitative chasmogamy

In wheat, spikelets are organized in spikes and proportion of chasmogamous to cleistogamous spikelets depend on genotype [6]. Considering the utilization of cleistogamy as a new strategy for controlling Fusarium head blight [15, 16, 69, 70], and physical preventing GM transmission [35, 71], cleistogamous plants should not release viable pollen at all. Extrusion of anthers even for part of spikelets will not prevent GMO transition and will pave a way for Fusarium infection [70]. Therefore, cleistogamy should be considered as a qualitative recessive trait [29] and chasmogamy as a quantitative trait related to anther extrusion [72–74]. Generally, the florets of rice, wheat, and barley are chasmogamous, but cleistogamy may be observed [29, 30, 32]. 14 genes were found in QTL regions for anther extrusion [7], but none one of them overlapped with the DEGs identified in our studies (Tables S1 and S3). This suggests that the mechanisms of lodicule driven florets opening and anther extrusion are determined by different genetic factors.

### Transcriptomic and proteomic mechanisms of flower opening

Spikelets of CH and CL wheat varieties at the GAS showed differential expression of genes associated with cell walls, carbohydrates, phytohormones, water channel, ion binding, and transport [16]. These genes regulate cellular homeostasis, osmotic pressure, and lodicule development [16] and may also be related to cold-stress, and drought-stress [75]. Just prior to lemma opening, CO_2_ accumulation and a drop in pH was reported in the lodicule cells [76] that results in the degradation of xyloglucan (XG) in the cell wall and promoting lodicule expansion [18].

The metabolic pathways involving the carbohydrates regulate the osmotic pressure of the lodicules [15]. In wheat, the proteomic changes associated with lodicule expanding mechanisms involved bidirectional sugar transporter, sucrose synthase, beta-amylase, as well as proteins related with cellular glucose homeostasis, and beta-glucosidase activity [15]. The accumulation of D-glucose leads to a change in osmotic pressure in the lodicules [12, 15, 17]. It was speculated that in lodicules of CL wheat (ZK001) the starch and total soluble sugar were metabolized via glycolysis and the tricarboxylic acid (TCA) cycle pathway while in the lodicules of CH wheat (YM18) starch and total soluble sugar content were enriched [16]. Five genes involved in carbohydrate metabolism were found within DEGs selected from RNA-Seq results. They belonged to 3 groups of vacuolar acid beta-fructofuranosidase (VIN), sucrose synthase, and UDP-D-glucose 4-epimerase (Table S6). The most out of 25 genes from RDA-Seq represented cell wall (CWIN) and vacuolar (VIN) acid beta-fructofuranosidases, sucrose synthases, and hexokinases (Table S7).

In wheat, the proteomic changes associated with lodicule expanding mechanisms involved annexin, calcium and potassium ion binding [15, 16]. Annexin can trigger calcium ion influx, increasing the osmotic pressure. Once the osmotic pressure changes, water accumulates in/is excreted from the cells of the lodicules and induces the expansion/shrinkage of the lodicules [15]. In barley aquaporin genes HvTIP1;1, HvTIP1;2, HvTIP2;3, and HvPIP2;1 coding tonoplast intrinsic proteins (TIPs) and plasma membrane intrinsic proteins (PIPs), were mostly upregulated in lodicules in the process of floret opening [36]. We colocalized the most (66 of 70) wheat aquaporins from TIPs, PIPs and nodulin 26-like intrinsic proteins Li et al. [36] with genes annotated at IWGSC RefSeq V1.0 genome, but no match was found for both RNA-Seq and RDA-Seq derived DEGs.

Metabolism of phytohormone and cell walls ensures lodicule swelling. Five kinds of plant endogenous hormones were reported to influence the opening of plant flowers, including ethylene, auxin, jasmonic, gibberellic and abscisic acids [77, 78]. In ears of CH barley (but not in CL ears), idole-3-acetic acid (IAA) and other synthetic auxins treatment resulted in bigger lodicules and flowering extended for a few days instead of hours. Treatment of CH plants with ABA or MJA reduced the numbers of exposed anthers [30]. In barley, anther was suggested to be a source of IAA [79] for lodicule enlargement [30]. In the lodicule of CL barley plants, physiological processes related to the transportation of, response to, or metabolism of auxin may not work well [30]. The flavin monooxygenases (FMOs) are key enzymes involved in rate-limiting step of tryptophan-dependent auxin biosynthesis [80, 81]. Expression level of a JA-related gene, allene oxidase synthase was upregulated in the lodicules of CH wheat YM18 from GAS to YAS [16]. In rice, JA-amino acid synthetase (OsJAR1) is required for optimal flower opening, closing and anther dehiscence [82]. Pathway Merkator analysis showed 5 phytohormone action DEGs identified with RNA-Seq analysis (Table S6). 36 DEGs were found in RDA-Seq analysis and these genes were related predominantly with jasmonic acid (14 genes), auxin (11 genes) and ethylene (2 genes) biosynthesis, signalling and degradation. Moreover, genes responsible for strigolactone biosynthesis, and signalling of gibberellin and cysteine-rich-peptide peptides were identified (Table S7). RT-qPCR assay revealed increased levels of expression in LCH cultivars in GAS for TraesCS2A02G141500, TraesCS5B02G344400, and TraesCS7A02G405600, with unknown function, involved in response to auxins, and flower development, respectively (Table S8, Fig. 10).

In CL sorghum, differentially expressed genes related with glycerolipid, glicerin metabolism, anthocyanin, phenylpropanoid biosynthesis, limonene and pinene degradation, and circadian rhythm were reported [40]. DEGs involved in secondary metabolites biosynthesis found in RDA-Seq analysis were mainly represented by mono-/sesquiterpene-/diterpene synthases [10], phenylalanine ammonia lyases [9] (Table S7). Single 3-hydroxy-3-methylglutaryl-CoA synthase (1), farnesyl diphosphate synthase (1), and chalcone synthase (1) were also found in this group. Beside chalcone synthase, RNA-Seq analysis also revealed pinoresinol/lariciresinol reductase (PLR) (Table S6).

We verified the RNA-Seq and RDA-Seq based expression profiles of 18 selected genes by RT-qPCR. Although the discussion on the legitimacy and reliability of such a verification is still ongoing and is not finally resolved, many authors (e.g. [43, 83, 84]) use this approach. Oppositely, Coenye [85] concluded that RNA-seq is robust enough and not always requires validation by qRT-PCR; nevertheless there are situations where confirmation of data obtained in large-scale transcriptomics may be of added value. This was the case in our study; RT-qPCR showed many discrepancies with the expression profiles determined by RNA-seq. These inconsistencies are primarily due to the different sensitivity of both methods. It should also be noted that the primers designed for RT-qPCR analysis could not be specific to the tested wheat cultivars. In case of our validation experiment the primers were not designed for random fragments of selected DEGs of Chinese Spring, but for the regions aligned with RDA-seq contigs. DNA sequences of the selected genes are not known in HCH and LCH cultivars and we targeted potentially unique regions of DEGs.

### Genetic determination of CL/CH

Flowers of most plant species develop according to ABCDE model which is based on tetramer complexes of different proteins specific for each whorl [86, 87]. Each protein complex is made up of MADS-domain transcription factors [86, 88] bound by class A, B, C, D, and E floral development-related proteins [89, 90]. In the ABCDE model, the identity of lodicules is mainly determined by B-class genes OsMADS2, OsMADS4, OsMADS6, OsMADS16 and OsMADS32, all of which encode transcription factors containing MADS-box domain [91–95].

Wheat orthologs of rice MIKC-type MADS-box genes involved in the ABCDE flowering model have been identified [89] and compared with DEGs found in our study (Tables S1 and S3). No hits were found for RNAseq data, but RDA-Seq analysis revealed four MADS genes differentially expressed. Orthologues of OsMADS16 (TraesCS7A02G383800 and TraesCS7B02G286600) were about 4–sixfold downregulated in CH cultivar Piko and Poezja vs Dacanto while wheat counterparts and OsMADS58 (TraesCS1A02G125800, and TraesCS1B02G144800) were sevenfold upregulated in Piko vs Dacanto (Table S3). RT-qPCR analysis provided no additional support for the role of wheat orthologues of OsMADS58 in shaping chasmogamy level in studied wheat cultivars (Fig. 10). However, two orthologues of OsMADS16 and TaMADS1 gene showed elevated expression in LCH cultivars in the GAS, that may contribute to chasmogamy differences in tested wheat cultivars. In rice CL afd1 and noh1 mutants, OsMADS16 mRNA levels were downregulated [28, 96]. OsMADS58 introduced into OsMADS58SRDX tall fescue showed a cleistogamous phenotype in which the lodicules were homeotically transformed into lemma-like organs [97]. Pathway analysis indicates that the expression level of wheat orthologues of MADS16 and MADS58 genes may be responsible for the quantitative differences in chasmogamy level in wheat. However, RT-qPCR results provide additional support only for MADS16.

In wheat, the simultaneous absence of both AP2L2 and AP2L5 genes results in spikelets with no lodicules and with other altered floral organs. In addition, AP2L2 affects lodicule size [89]. In studies of diverse set of wheat germplasm, covering a range of ploidy levels and ranging from cultivated varieties to wild relatives, the plants were uniformly non-cleistogamous, and their AP2 homoeologous sequences were highly conserved [19]. In our study, the expression of AP2-like genes was not significantly different between LCH and HCH wheat cultivars.

## Conclusions

The presented results show that the lodicules played an important role in the flowering process in wheat. Furthermore, our data indicate that the expression level of *MADS16* and *MADS58* genes are responsible for the quantitative differences in chasmogamy level in wheat. Moreover, the genes responsible for phytohormones biosynthesis, signalling and degradation were reported to influence the opening of plant flowers.

Based on GO enrichment analysis, nine DEGs upregulated in highly-chasmogamous cultivars were involved in stamen filament development indicating significance of those genes in flowering. We conclude that the DEGs identified in our study with a high and stable expression in cultivars with different intensity of chasmogamy are good candidates responsible for lodicule mediated flower opening. Combining and utilising the results from this and our previous study [43] can ensure progress in wheat hybrid breeding programs.

## Supplementary Information

**Additional file 1: Figure S1.** Agri GO analysis of DEGs upregulated in category of biological process.

**Additional file 2:** **Table S1.** Fold changes of 594 down- and upregulated DEGs at the GAS and YAS of HCH (Pi-Piko, Po-Poezja) vs LCH (Eu-Euforia, Da-KWS Dacanto) cultivars identified in RNA-Seq analysis. **Table S2.** Expression levels of selected DEGs during Chinese Spring development (http://wheatomics.sdau.edu.cn/expression/wheat.html). **Table S3.** List of 1161 DEGs down- and upregulated at lodicules at the GAS and YAS of HCH (Pi-Piko, Po-Poezja) vs LCH (Eu-Euforia, Da-KWS Dacanto) cultivars identified in RDA-Seq analysis. **Table S4.** DEGs selected in RNA-seq and RDA-seq analyses, their sequences, location, promoter regions, and the best blast hits. **Table S5.** Regulatory elements identified in regions 2,000 bp upstream of selected DEGs. **Table S6.** DEGs identified in RNA-Seq analysis annotated in Merkator (https://www.plabipd.de/portal/mercator4) database. **Table S7.** DEGs identified in RDA-Seq analysis annotated in Merkator (https://www.plabipd.de/portal/mercator4) database. **Table S8.** Characteristics of 18 genes selected for qPCR analysis and PCR primers.

## Data Availability

The datasets generated and/or analysed during the current study are available in the ArrayExpress database at EMBL-EBI (www.ebi.ac.uk/arrayexpress) under accession number E-MTAB-12136 (RNA-seq data) and E-MTAB-12139 (RDA-seq data).

## Abbreviations

BP: Biological Process
DEG: Differentially Expressed Gene
FDR: False Discovery Rate
FPKMFragments Per: Kilobase of sequence per Million mapped reads
GAS: Green Anther Stage
GO: Gene Ontology
HCH: Highly-Chasmogamous Cultivars
KEGGKyoto: Encyclopedia of Genes and Genomes
LCH: Low-Chasmogamy Cultivars
MF: Molecular Function
RDARepresentational: Difference Analysis
SEA: Singular Enrichment Analysis
YAS: Yellow Anther Stage

## References

[1] De Vries AP (1971). Flowering biology of wheat, particularly in view of hybrid seed production—a review. Euphytica.

[2] Waddington S, Cartwright P, Wall P (1983). A quantitative scale of spike initial and pistil development in barley and wheat. Ann Bot.

[3] Ueno K, Itoh H (1997). Cleistogamy in wheat: genetic control and the effect of environmental conditions. Cereal Res Com.

[4] Langer SM, Longin CFH, Würschum T. Phenotypic evaluation of floral and flowering traits with relevance for hybrid breeding in wheat (*Triticum aestivum* L.). Plant Breed. 2014; doi:10.1111/pbr.12192.

[5] Okada T, Jayasinghe J, Nansamba M, Baes M, Warner P, Kouidri A, Correia D, Nguyen V, Whitford R, Baumann U (2018). Unfertilized ovary pushes wheat flower open for cross- pollination. J Exp Bot.

[6] Zajączkowska U, Denisow B, Łotocka B, Dołkin-Lewko A, Rakoczy-Trojanowska M (2021). Spikelet movements anther extrusion and pollen production in wheat cultivars with contrasting tendencies to cleistogamy. BMC Plant Biol.

[7] Muqaddasi QH, Jayakodi M, Börner A, Röder MS (2019). Identification of consistent QTL with large effect on anther extrusion in doubled haploid populations developed from spring wheat accessions in German Federal ex situ Genebank. Theor Appl Genet.

[8] Zee S, O’Brien T (1971). The vascular tissue of the lodicules of wheat. Aust J Biol Sci.

[9] Frankel R, Galun E (1977). Pollination mechanisms, reproduction and plant breeding.. Autogamy..

[10] Virmani SS (1994). Outcrossing mechanisms and hybrid seed production practices in rice.. Heterosis and hybrid rice breeding..

[11] Yoshida H (2012). Is the lodicule a petal: molecular evidence?. Plant Sci.

[12] Heslop-Harrison YJS, Heslop-Harrison JS (1996). Lodicule function and filament extension in the grasses: potassium ion movement and tissue specialization. Ann Bot.

[13] Kirby EJM, Fellowes G, Appleyard M (1983). Anthesis in winter barley.. Annual report—Plant Breeding Institute..

[14] Pickett A (1993). Hybrid wheat results and problems. Portschritte der Pflanzenzüchtung..

[15] Tang C, Zhang H, Zhang P, Ma Y, Cao M, Hu H, Shah FA, Zhao W, Li M, Wu L (2019). iTRAQ-based quantitative proteome analysis reveals metabolic changes between a cleistogamous wheat mutant and its wild-type wheat counterpart. PeerJ.

[16] Tang C, Li M, Cao M, Lu R, Zhang H, Liu C, Huang S, Zhang P, Hu H, Zhao W, Wu L. Transcriptome analysis suggests mechanisms for a novel flowering type: Cleistogamous wheat. Crop J. 2020; doi:10.1016/j.cj.2019.08.009.

[17] Liu L, Zou ZS, Qian K, Xia C, He Y, Zeng HL, Zhou X, Riemann M, Yin CX (2017). Jasmonic acid deficiency leads to scattered floret opening time in cytoplasmic male sterile rice Zhenshan 97A. J Exp Bot.

[18] Qin Y, Yang J, Zhao J (2005). Calcium changes and the response to methyl jasmonate in rice lodicules during anthesis Protoplasma.

[19] Ning S, Wang N, Sakuma S, Pourkheirandish M, Koba T, Komatsuda T (2013). Variation in the wheat AP2 homoeologs, the genes underlying lodicule development. Breed Sci.

[20] Yuan G, Wang Y, Yuan S, Wang P, Duan W, Bai J, Sun H, Wang N, Zhang F, Zhang L, Zhao C (2018). Functional analysis of wheat *TaPaO1* gene conferring pollen sterility under low temperature. J Plant Biol.

[21] Pandey B, Sharma P, Pandey DM, Sharma I, Chatrath R (2013). Identification of new aquaporin genes and single nucleotide polymorphism in bread wheat. Evol Bioinform.

[22] Peng FY, Hu Z, Yang RC (2015). Genome-wide comparative analysis of flowering-related genes in arabidopsis, wheat, and barley. Int J Plant.

[23] Lord E (1981). Cleistogamy: a tool for the study of floral morphogenesis, function and evolution. Bot Rev.

[24] Hirano HY, Tanaka W, Toriba T (2014). Grass flower development. Methods Mol Biol.

[25] Smoczynska A, Szweykowska-Kulinska Z (2016). MicroRNA-mediated regulation of flower development in grasses. Acta Biochim Pol.

[26] Nair SK, Wang N, Turuspekov Y, Pourkheirandish M, Sinsuwongwat S, Chen G, Sameri M, Tagiri A, Honda I, Watanabe Y, Kanamori H, Wicker T, Stein N, Nagamura Y, Matsumoto T, Komatsuda T (2009). Cleistogamous flowering in barley arises from the suppression of microRNA-guided *HvAP2* mRNA cleavage. Proc Natl Acad Sci U S A.

[27] Lombardo F, Kuroki M, Yao SG, Shimizu H, Ikegaya T, Kimizu M, Ohmori S, Akiyama T, Hayashi T, Yamaguchi T, Koike S, Yatou O, Yoshida H (2017). The superwoman1-cleistogamy2 mutant is a novel resource for gene containment in rice. Plant Biotechnol J.

[28] Zhang J, Zheng H, Zeng X, Zhuang H, Wang H, Tang J, Chen H, Ling Y, Li Y. Characterization and gene mapping of non-open hull 1 (noh1) mutant in rice (*Oryza sativa* L.). Agronomy. 2019; 10.3390/agronomy9020056.

[29] Chhabra AK, Sethi SK (1991). Inheritance of cleistogamic flowering in durum wheat (*Triticum durum*). Euphytica.

[30] Honda I, Turuspekov Y, Komatsuda T, Watanabe Y (2005). Morphological and physiological analysis of cleistogamy in barley (*Hordeum vulgare*). Physiol Plant.

[31] He X, Singh PK, Dreisigacker S, Singh S, Lillemo M, Duveiller E (2016). Dwarfing genes *Rht-B1b* and *Rht-D1b* are associated with both type I FHB susceptibility and low anther extrusion in two bread wheat populations. PLoS ONE.

[32] Wu F, Zhang D, Muvunyi BP, Yan Q, Zhang Y, Yan Z, Cao M, Wang Y, Zhang J (2018). Analysis of microRNA reveals cleistogamous and chasmogamous floret divergence in dimorphic plant. Sci Rep.

[33] Wang N, Ning S, Pourkheirandish M, Honda I, Komatsuda T (2013). An alternative mechanism for cleistogamy in barley. Theor Appl Genet.

[34] Ning S, Wang N, Sakuma S, Pourkheirandish M, Wu J, Matsumoto T, Koba T, Komatsuda T (2013). Structure, transcription and post-transcriptional regulation of the bread wheat orthologs of the barley cleistogamy gene Cly1. Theor Appl Genet.

[35] Ni DH, Li J, Duan YB, Yang YC, Wei PC, Xu RF, Li CR, Liang DD, Li H, Song FS, Ni JL, Li L, Yang JB. Identification and utilization of cleistogamy gene cl7(t) in rice (*Oryza sativa* L.). J Exp Bot. 2014; 10.1093/jxb/eru074. 10.1093/jxb/eru074 24619999

[36] Li Q, Tong T, Jiang W, Cheng J, Deng F, Wu X, Chen Z-H, Ouyang Y, Zeng F (2022). Highly conserved evolution of aquaporin PIPs and TIPs confers their crucial contribution to flowering process in plants. Front Plant Sci.

[37] Chen Y, Ma J, Miller AJ, Luo B, Wang M, Zhu Z, Ouwerkerk PB (2016). OsCHX14 is involved in the K+ homeostasis in rice (*Oryza sativa*) flowers. Plant Cell Physiol.

[38] Zhong W, Yunjie G, Yuzhu G. Studies on the mechanism of the anthesis of rice III. Structure of the lodicule and changes of its contents during flowering. Acta Agron Sin. 1991;17:96–101.

[39] Li X, Wang Y, Duan E (2018). OPEN GLUME1: a key enzyme reducing the precursor of JA, participates in carbohydrate transport of lodicules during anthesis in rice. Plant Cell Rep.

[40] Liu S, Fu Y, He Y, Zeng X (2021). Transcriptome analysis of the impact of exogenous methyl jasmonate on the opening of sorghum florets. PLoS ONE.

[41] Chen J, Xu Y, Fei K, Wang R, He J, Fu L, Chen J, Xu Y, Fei K, Wang R, He J, Fu L, Shao S, Li K, Zhu K, Zhang W, Wang Z, Yang J (2020). Physiological mechanism underlying the effect of high temperature during anthesis on spikelet-opening of photo-thermo-sensitive genic male sterile rice lines. Sci Rep.

[42] Wang N, Ning S, Wu J, Tagiri A, Komatsuda T (2015). An epiallele at cly1 affects the expression of floret closing (cleistogamy) in barley. Genetics.

[43] Tyrka M, Bakera B, Szeliga M, Święcicka M, Krajewski P, Mokrzycka M, Rakoczy-Trojanowska M. Identification of *Rf* Genes in hexaploid wheat (*Triticum aestivum* L.) by RNA-Seq and Paralog analyses. Int J Mol Sci. 2021; 10.3390/ijms22179146. 10.3390/ijms22179146 PMC8431562 34502055

[44] Trapnell C, Roberts A, Goff L, Pertea G, Kim D, Kelley DR, Pimentel H, Salzberg SL, Rinn JL, Pachter L (2012). Differential gene and transcript expression analysis of RNA-seq experiments with TopHat and Cufflinks. Nat Protoc.

[45] Anders S, Pyl PT, Huber W (2014). HTSeq — A Python framework to work with highthroughput sequencing data. Bioinformatics.

[46] The Gene Ontology Consortium (2000). Gene Ontology: tool for the unification of biology. Nature Genet.

[47] Tian T, Liu Y, Yan H, You Q, Yi X, Du Z, Xu W, Su Z. agriGO v2.0: a GO analysis toolkit for the agricultural community, 2017 update. Nucleic Acids Res. 2017; 10.1093/nar/gkx382. 10.1093/nar/gkx382 PMC5793732 28472432

[48] Moriya Y, Itoh M, Okuda S, Yoshizawa AC, Kanehisa M (2007). KAAS: an automatic genome annotation and pathway reconstruction server. Nucleic Acids Res.

[49] Bardou P, Mariette J, Escudié F, Djemiel Ch, Klopp Ch. jvenn: an interactive Venn diagram viewer. BMC Bioinform. 2014; 10.1186/1471-2105-15-293. 10.1186/1471-2105-15-293 PMC4261873 25176396

[50] Babicki S, Arndt D, Marcu A, Liang Y, Grant R, Maciejewski A, Wishart DS (2016). Heatmapper: web-enabled heat mapping for all. Nucleic Acids Res.

[51] Ciura J, Szeliga M, Grzesik M, Tyrka M (2017). Next-generation sequencing of representational difference analysis products for identification of genes involved in diosgenin biosynthesis in fenugreek (*Trigonella foenum-graecum*). Planta.

[52] Szeliga M, Ciura J, Tyrka M (2020). Representational difference analysis of transcripts involved in jervine biosynthesis. Life (Basel).

[53] Schwacke R, Ponce-Soto GY, Krause K, Bolger AM, Arsova B, Hallab A, Gruden K, Stitt M, Bolger ME, Usadel B (2019). MapMan4: a refined protein classification and annotation framework applicable to multi-omics data analysis. Mol Plant.

[54] Ma S, Wang M, Wu J, Guo W, Chen Y, Li G, Wang Y, Shi W, Xia G, Fu D, Kang Z, Ni F (2021). WheatOmics: a platform combining multiple omics data to accelerate functional genomics studies in wheat. Mol Plant.

[55] Hou K, Wu W, Gan SS (2013). SAUR36, a small auxin up RNA gene, is involved in the promotion of leaf senescence in *Arabidopsis*. Plant Physiol.

[56] Stamm P, Kumar PP. Auxin and gibberellin responsive Arabidopsis SMALL AUXIN UP RNA36 regulates hypocotyl elongation in the light. Plant Cell Rep. 2013; 10.1007/s00299-013-1406-5. 10.1007/s00299-013-1406-5 23503980

[57] Borghi L, Kang J, Ko D, Lee Y, Martinoia E (2015). The role of ABCG-type ABC transporters in phytohormone transport. Biochem Soc Trans.

[58] Strader LC, Bartel B (2009). The Arabidopsis PLEIOTROPIC DRUG RESISTANCE8/ABCG36 ATP binding cassette transporter modulates sensitivity to the auxin precursor indole-3-butyric acid. Plant Cell.

[59] Ziegler J, Schmidt S, Strehmel N, Scheel D, Abel S (2017). *Arabidopsis* transporter ABCG37/PDR9 contributes primarily highly oxygenated coumarins to root exudation. Sci Rep.

[60] Ruzicka K, Strader LC, Bailly A, Yang H, Blakeslee J, Langowski L, Nejedlá E, Fujita H, Itoh H, Syono K, Hejátko J, Gray WM, Martinoia E, Geisler M, Bartel B, Murphy AS, Friml J (2010). *Arabidopsis* PIS1 encodes the ABCG37 transporter of auxinic compounds including the auxin precursor indole-3-butyric acid. Proc Natl Acad Sci U S A.

[61] Guimier A, Gordon CT, Godard F, Ravenscroft G, Oufadem M, Vasnier C, Rambaud C, Nitschke P, Bole-Feysot C, Masson C, Dauger S, Longman C, Laing NG, Kugener B, Bonnet D, Bouvagnet P, Di Filippo S, Probst V, Redon R, Charron P, Rötig A, Lyonnet S, Dautant A, de Pontual L, di Rago JP, Delahodde A, Amiel J (2016). Biallelic PPA2 mutations cause sudden unexpected cardiac arrest in infancy. Am J Hum Genet.

[62] Larsen AK, Skladanowski A, Bojanowski K (1996). The roles of DNA topoisomerase II during the cell cycle. Prog Cell Cycle Res.

[63] Han G, Lu C, Guo J, Qiao Z, Sui N, Qiu N, Wang B (2020). C2H2 Zinc finger proteins: master regulators of abiotic stress responses in plants. Front Plant Sci.

[64] Peng YL, Shirano Y, Ohta H, Hibino T, Tanaka K, Shibata D. A novel lipoxygenase from rice. Primary structure and specific expression upon incompatible infection with rice blast fungus. J Biol Chem. 1994; 10.1016/S0021-9258(17)41924-7. 7508918

[65] Grienenberger E, Kim SS, Lallemand B, Geoffroy P, Heintz D, Souza Cde A, Heitz T, Douglas CJ, Legrand M (2010). Analysis of TETRAKETIDE α-PYRONE REDUCTASE function in *Arabidopsis thaliana* reveals a previously unknown, but conserved, biochemical pathway in sporopollenin monomer biosynthesis. Plant Cell.

[66] Duan E, Wang Y, Li X, Lin Q, Zhang T, Wang Y, Zhou C, Zhang H, Jiang L, Wang J, Lei C, Zhang X, Guo X, Wang H, Wan J (2019). OsSHI1 regulates plant architecture through modulating the transcriptional activity of IPA1 in rice. Plant Cell.

[67] Schaller F, Biesgen C, Müssig C, Altmann T, Weiler EW (2000). 12-Oxophytodienoate reductase 3 (OPR3) is the isoenzyme involved in jasmonate biosynthesis. Planta.

[68] Sedbrook JC, Carroll KL, Hung KF, Masson PH, Somerville CR (2002). The *Arabidopsis SKU5* gene encodes an extracellular glycosyl phosphatidylinositol-anchored glycoprotein involved in directional root growth. Plant Cell.

[69] Kubo K, Kawada N, Fujita M, Hatta K, Oda S, Nakajima T (2010). Effect of cleistogamy on *Fusarium* head blight resistance in wheat. Breed Sci.

[70] Herrmann MH, Hautsalo J, Georgieva P, Bund A, Winter M, Beuch S (2020). Relationship between genetic variability of flowering traits and *Fusarium* mycotoxin contamination in oats. Crop Sci.

[71] Ohmori S, Koike S, Hayashi T, Yamaguchi T, Kuroki M, Yoshida H (2018). The cleistogamy of the *superwoman1-cleistogamy1* mutation is sensitive to low temperatures during the lodicule-forming stage. Breed Sci.

[72] Muqaddasi QH, Lohwasser U, Nagel M, Börner A, Pillen K, Röder MS (2016). Genome-wide association mapping of anther extrusion in hexaploid spring wheat. PLoS ONE.

[73] Muqaddasi QH, Brassac J, Borner A, Pillen K, Roder MS (2017). Genetic architecture of anther extrusion in spring and winter wheat. Front Plant Sci.

[74] Adhikari A, Basnet BR, Crossa J, Dreisigacker S, Camarillo F, Bhati PK, Jarquin D, Manes Y, Ibrahim AMH (2020). Genome-wide association mapping and genomic prediction of anther extrusion in CIMMYT hybrid wheat breeding program via modeling pedigree, genomic relationship, and interaction with the environment. Front Genet.

[75] Morinaga SI, Nagano AJ, Miyazaki S, Kubo M, Demura T, Fukuda H, Sakai S, Hasebe M (2008). Ecogenomics of cleistogamous and chasmogamous flowering: genome-wide gene expression patterns from cross-species microarray analysis in *Cardamine kokaiensis* (Brassicaceae). J Ecol.

[76] Jin YG, Zhao BH, Wang WH, Ge CL, Li HY. Changes of structure and substances in the lodicule during the opening and closing lemma of barley. Acta Bot Boreal-Occid Sin. 1998;18:595-601.

[77] Santner A, Calderon-Villalobos LI, Estelle M (2009). Plant hormones are versatile chemical regulators of plant growth. Nat Chem Biol.

[78] Wang YH, Irving HR (2011). Developing a model of plant hormone interactions. Plant Signal Behav.

[79] Koning RE (1983). The role of auxin, ethylene, and acid growth in filament elongation in *Gaillardia grandiflora* (Asteraceae). Am J Bot.

[80] Zhao Y, Christensen SK, Fankhauser C, Cashman JR, Cohen JD, Weigel D (2001). A role for flavin monooxygenase-like enzymes in auxin biosynthesis. Science.

[81] Chen J, Somta P, Chen X, Cui X, Yuan X and Srinives P. Gene mapping of a mutant mungbean (*Vigna radiata* L.) Using new molecular markers suggests a gene encoding a YUC4-like protein regulates the chasmogamous flower trait. Front Plant Sci. 2016b; 10.3389/fpls.2016.00830. 10.3389/fpls.2016.00830 PMC4901043 27375671

[82] Xiao Y, Chen Y, Charnikhova T, Mulder PP, Heijmans J, Hoogenboom A, Agalou A, Michel C, Morel JB, Dreni L, Kater MM, Bouwmeester H, Wang M, Zhu Z, Ouwerkerk PB (2014). *OsJAR1* is required for JA-regulated floret opening and anther dehiscence in rice. Plant Mol Biol.

[83] Yim AKY, Wong JWH, Ku YS, Qin H, Chan TF, Lam HM (2015). Using RNA-Seq Data to Evaluate Reference Genes Suitable for Gene Expression Studies in Soybean. PLoS ONE..

[84] Zeng F, Biligetu B, Coulman B, Schellenberg MP, Fu Y-B (2017). RNA-Seq analysis of gene expression for floral development in crested wheatgrass (Agropyron cristatum L.). PLoS ONE..

[85] Coenye T (2021). Do results obtained with RNA-sequencing require independent verification?. Biofilm..

[86] Theißen G, Saedler H (2001). Plant biology-floral quartets. Nature.

[87] Theißen G, Melzer R, Rumpler F (2016). MADS-domain transcription factors and the floral quartet model of flower development: linking plant development and evolution. Development.

[88] Coen E, Meyerowitz EM (1991). The war of the whorls: genetic interactions controlling flower development. Nature.

[89] Pelaz S, Ditta GS, Baumann E, Wisman E, Yanofsky MF (2000). B and C floral organ identity functions require SEPALLATA MADS-box genes. Nature.

[90] Debernardi JM, Greenwood JR, Jean Finnegan E, Jernstedt J, Dubcovsky J (2020). APETALA 2-like genes AP2L2 and Q specify lemma identity and axillary floral meristem development in wheat. Plant J.

[91] Xiao H, Wang Y, Liu D, Wang W, Li X, Zhao X, Xu J, Zhai W, Zhu L (2003). Functional analysis of the rice AP3 homologue *OsMADS16* by RNA interference. Plant Mol Biol.

[92] Yadav SR, Prasad K, Vijayraghavan U (2007). Divergent regulatory *OsMADS2* functions control size shape and differentiation of the highly derived rice floret second-whorl organ. Genetics.

[93] Yao SG, Ohmori S, Kimizu M, Yoshida H (2008). Unequal genetic redundancy of rice PISTILLATA orthologs, *OsMADS2* and *Os-MADS4*, in lodicule and stamen development. Plant Cell Physiol.

[94] Li H, Liang W, Jia R, Yin C, Zong J, Kong H, Zhang D (2010). The AGL6-like gene *OsMADS6* regulates floral organ and meristem identities in rice. Cell Res.

[95] Wang H, Zhang L, Cai Q, Hu Y, Jin Z, Zhao X, Fan W, Huang Q, Luo Z, Chen M, Zhang D, Yuan Z (2015). OsMADS32 interacts with PI - like proteins and regulates rice flower development. J Integr Plant Biol.

[96] Ren D, Rao Y, Wu L, Xu Q, Li Z, Yu H, Zhang Y, Leng Y, Hu J, Zhu L, Gao Z, Dong G, Zhang G, Guo L, Zeng D, Qian Q (2016). The pleiotropic ABNORMAL FLOWER AND DWARF1 affects plant height, fl oral development and grain yield in rice. J Integr Plant Biol.

[97] Sato H, Yoshida K, Mitsuda N, Ohme-Takagi M, Takamizo T (2012). Male-sterile and cleistogamous phenotypes in tall fescue induced by chimeric repressors of SUPERWOMAN1 and OsMADS58. Plant Sci.

