# Supplementary figures and images for: Identification of candidate genes responsible for chasmogamy in wheat
Source: BMC Genomics. 2023 Apr 4;24:170. doi: 10.1186/s12864-023-09252-1 (PMC10074802; doi:10.1186/s12864-023-09252-1)

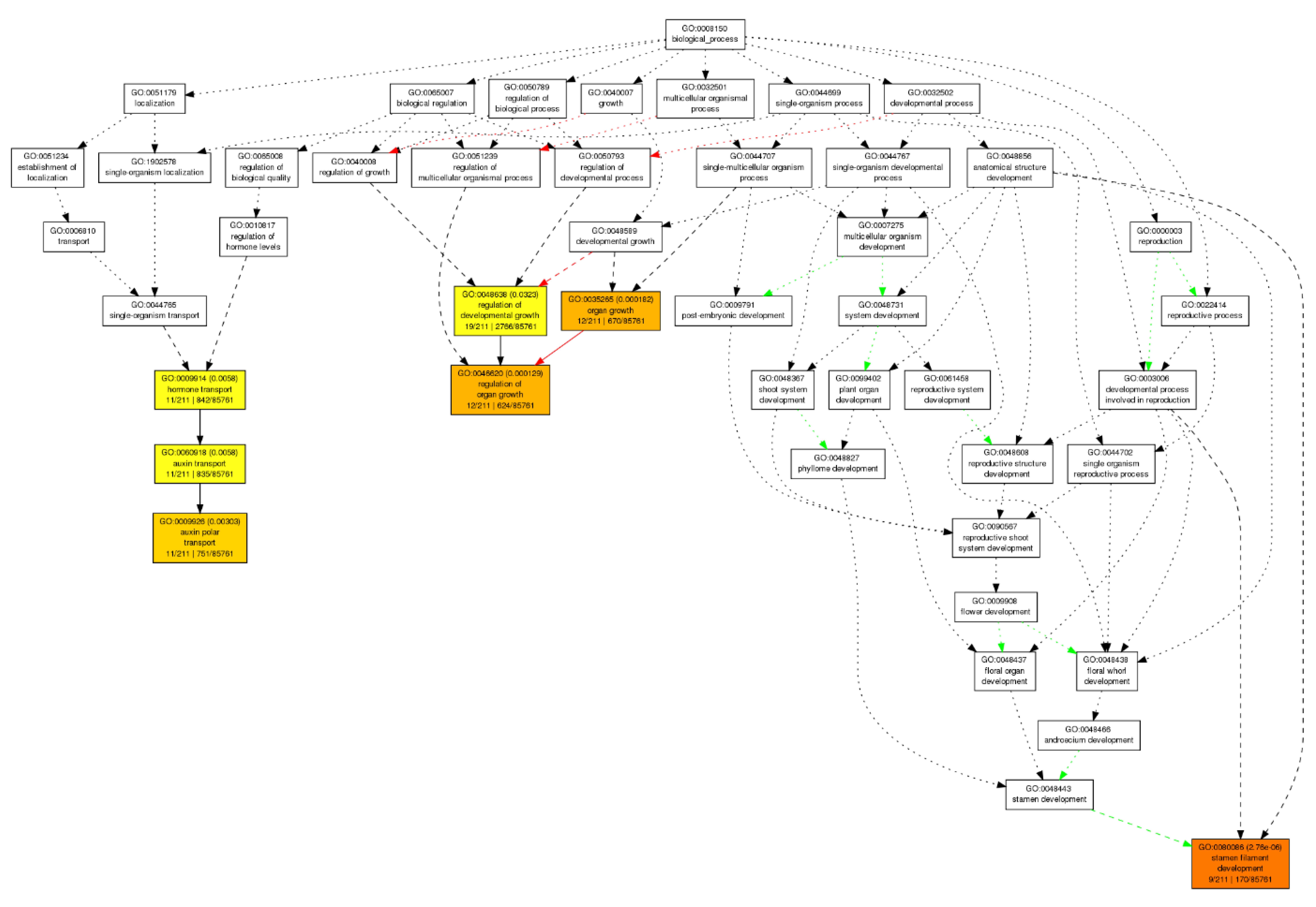

Supplement: Supplementary file 1 — Additional file 1: Figure S1. Agri GO analysis of DEGs upregulated in category of biological process. [file 12864_2023_9252_MOESM1_ESM.jpg]
